# Outcomes and Acceptability of the Community‐Based Occupational Well‐Being Intervention Among Health Care Educators—Mixed Method Pilot Study

**DOI:** 10.1111/scs.70161

**Published:** 2025-12-10

**Authors:** Anneli Vauhkonen, Kirsi Honkalampi, Jenni Rinne, Leena Salminen, Terhi Saaranen

**Affiliations:** ^1^ Department of Nursing Science University of Eastern Finland Kuopio Finland; ^2^ Philosophical Faculty, School of Educational Sciences and Psychology University of Eastern Finland Joensuu Finland; ^3^ City of Turku Turku Finland; ^4^ Department of Nursing Science University of Turku Turku Finland; ^5^ Turku University Hospital Turku Finland

**Keywords:** acceptability, educator, health care, intervention, mixed method, occupational well‐being

## Abstract

**Aims and Objectives:**

To evaluate the outcomes and acceptability of the Community‐based Participatory Occupational Well‐being Intervention for Educators.

**Methodological Design and Justification:**

This pilot study used a sequential explanatory mixed‐method intervention study design in which the post‐intervention qualitative data were embedded in the quasi‐experimental pre‐test–post‐test data to explain and expand the intervention outcomes and to evaluate intervention acceptability.

**Ethical Issues and Approval:**

This study received an ethical statement from the UEF Committee on Research Ethics (7/2021 15.4.2021) and followed the ethical principles of the Declaration of Helsinki.

**Research Methods and Intervention:**

The quantitative pre‐and post‐test data were collected using an electronic questionnaire among health care educators (intervention *n* = 19, comparison *n* = 22). The 1‐year intervention included (i) an online course, formation of (ii) an occupational well‐being development team, which (iii) planned and (iv) implemented community‐specific development actions. Qualitative interview data were collected from participants (*n* = 9) 3 months post‐intervention. The quantitative data were analysed statistically and the qualitative data by deductive–inductive content analysis. The main results were merged into a joint display as mixed‐method meta‐inferences.

**Results:**

Positive changes were found in occupational well‐being, promoting activities and workplace support. Educators experienced improvements in work organisation processes and reflection on occupational well‐being issues. The study found no significant change in the overall self‐assessed level of occupational well‐being. The intervention framework was considered functional, with workload issues as the main barriers.

**Study Limitations:**

The main limitations of this study were a small sample size and a long intervention period, which challenged participant engagement and outcome evaluation.

**Conclusions:**

The intervention enables community‐level occupational well‐being development, and it can be applied in health care educators' work communities. The study suggests refining the intervention in terms of information provision, time resources and community‐level orientation and discussion.

## 1 Background

Maintaining occupational well‐being (hereafter OW) of health care educators is one of the ways to respond to the growing need for educators working in health care education [1, 2], and is vital for the well‐being of the students [3]. As working careers lengthen and the population ages, it is important to maintain and promote the OW of the already existing workforce, as well as increase the job attraction and retention power of the sector [2]. Health care educators perceive their work as valuable due to the autonomy it provides, their meaningful role in facilitating student success, and the flexible nature of their professional responsibilities [4, 5, 6]. However, various responsibilities beyond teaching—such as research and project work, curriculum development, leadership and administrative duties within the institution, often involving emerging digital health solutions and online technologies [2], can create considerable mental demands [7]. In addition to these demands, health care educators often face high workload and backlog situations [7, 8], work‐life imbalance [7, 9, 10], and challenges in the work community, including limited resources, lack of support, unequal treatment and management issues [5, 11, 12]. These factors can decrease the well‐being of educators and increase the risk for work‐related stress [11], burnout [13], and the intent to leave the profession [2, 9]. Although studies have established the need for OW intervention research [8, 14], intervention research aimed at promoting the OW of health care educators is scarce [15, 16, 17, 18, 19].

This study applies a resource‐based perspective for OW based on a Content Model for the Promotion of School Community Staff's OW (Content Model ProSchoolSOWE) [20, 21]. The model defines OW through four aspects: (1) Working conditions (e.g., physical working environment), (2) Work community (e.g., work management and organisation, social support), (3) Worker's resources and work (e.g., mental and physical workload) and (4) Professional competence (e.g., professional competence and educational opportunities) [20, 21, 22]. It has been used as a framework for both individual [16], and community‐level OW interventions [22, 23].

Community‐level OW interventions can lead to positive, long‐term changes in work communities by addressing the actual causes of decreased OW [24]. It is a theory‐based action aimed at improving health and well‐being by changing how work is organised, designed and managed [25]. Emphasising the Culture of Health (COH) in the workplace. COH is an environment that values and promotes employee health and well‐being [26, 27], and is positively related to employee health, engagement, retention and organisational success [27]. Community‐level OW interventions often use participatory approaches, involving participants in planning and decision‐making [23, 28], which increases readiness for change and intervention engagement [25].

Community‐level interventions have sub‐objectives that create a chain of positive effects, starting from attitude change and leading to improved work processes and improved well‐being [25]. These complex interventions involve multiple components and processes, with difficulty detecting changes requiring a wide range of assessment methods [29]. Thus, it is crucial to evaluate the process and acceptance: how the intervention is accepted by the users; how it works and under what circumstances [29, 30, 31]. Acceptability can be defined as participants' cognitive and emotional responses, and stakeholders' views on the intervention's relevance and applicability, such as usability and utility in a real‐life setting [32, 33]. Perski et al. [34], also link engagement to the acceptability concept, which is essential to the successful implementation of participatory interventions.

Implementation of community‐level OW interventions is scarce, and their acceptability has been infrequently evaluated in health care education. A year‐long gratitude intervention in the work community found positive outcomes on job satisfaction [18]. A workplace wellness online learning tool increased workplace wellness knowledge and was evaluated as usable and engaging, with positive user attitudes [15]. A digital OW intervention using self‐conducted exercises for nurse educators found positive effects on the educators' well‐being; it was also found to be applicable to use together with colleagues and students in their daily work routines [16, 17]. This study adds knowledge to this scarcely studied research context, and therefore, aims to evaluate the outcomes and acceptability of the Community‐based Participatory OW Intervention for Educators (CBP‐OWE) among health care educators. Research questions were formulated:

1. What are the outcomes of the CBP‐OWE intervention based on:
  - The pre‐ and post‐test outcomes on within‐ and between‐group comparison?
  - interviewed participants experiences of the intervention relevance on OW?
  - the interview experiences explaining and expanding the pre‐ and post‐test outcomes?
2. How did participants experience the acceptability of the intervention?

## 2 Methods

### 2.1 Study Design and Setting

This sequential explanatory mixed‐method CBP‐OWE intervention pilot study contained two sub‐studies and three measurement points: (1) The outcomes evaluation was a quasi‐experimental pre‐ and post‐test (T0, T1) study with qualitative interview data embedded 3 months post‐intervention (T2); (2) The acceptability of the intervention was assessed through qualitative interviews (T2). Health care educators from health care programmes at one university of applied sciences participated in the intervention, while another university of applied sciences had the comparison group. The universities provided educational programmes in nursing, public health nursing, midwifery and paramedic nursing. The (TiDieR) Template for Intervention Description and Replication Checklist [35], and Mixed Methods Reporting in Rehabilitation & Health Sciences (MMR‐RHS) [36], were both applied when reporting the study.

### 2.2 Intervention

The research group created the CBP‐OWE intervention based on previous literature on OW in health care education, and on the results of a cross‐sectional study [5, 8], and existing university OW educational courses. The course was tested by four university educators. The main components of the CBP‐OWE intervention are: (i) an OW development team (OWE team), (ii) an OW for Educators (OWE‐Edu) course, (iii) an OW development plan and (iv) OW actions. The intervention content and expected functionality [30], are presented in the Figure S1.

**FIGURE 1:**
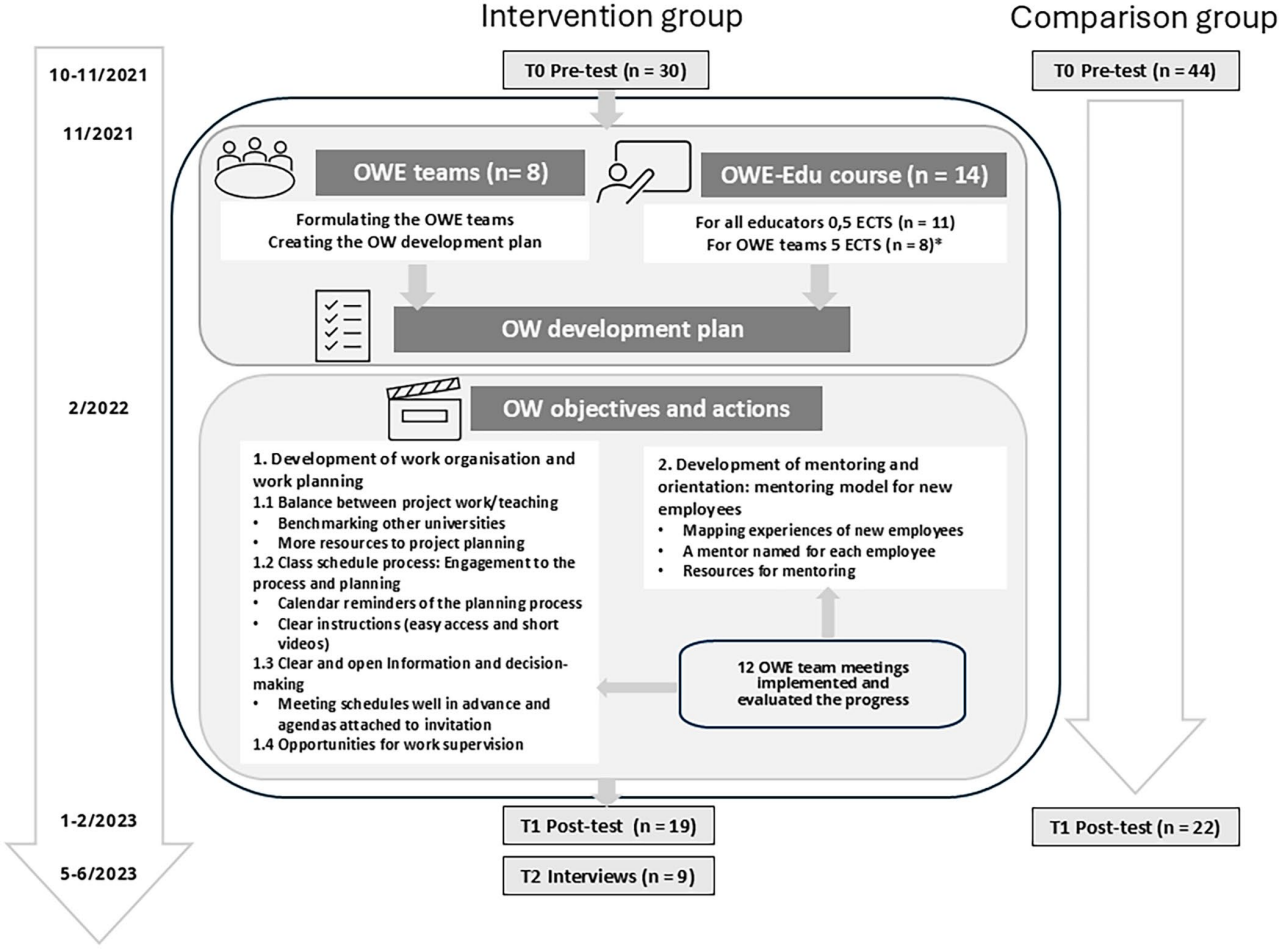
Implementation and the study design of the CBP‐OWE intervention. OW, occupational well‐being; OWE‐Edu course, Occupational Well‐being for Educators course; OWE team, Occupational Well‐being development team; *5 OWE‐team members also participated in the 0,5 ECTS course (with permission from the authors).

The CBP‐OWE intervention was implemented collaboratively with the main researcher and the work community and lasted 14 months (Figure 1), following a cyclical action research process of planning, action, observation and reflection phases description of the actions research process in Vauhkonen et al. [37]. Due to the low participation, the OWE‐Edu course was kept open for the whole intervention period rather than the originally planned 1 month. The OWE team was formulated, comprising an educator from all working teams at the work community and a direct supervisor (*n* = 8 members). Pre‐test results were discussed in face‐to‐face meetings and an online workshop, leading to an OW development plan focused on work organisation and planning, and mentoring to balance educators' mental workload and improve their own work management. The plan was refined mid‐term to include work supervision opportunities. Post‐test results were shared with the work community, along with future development recommendations.

### 2.3 Data Collection Methods

The study used the validated OW of Social and Health Care Teachers instrument (OWESoHeT) [38], of which four items measured overall OW and OW promoting activities (continuous scale 0–5, 0 = very poor, 5 = very good) and 29 items covered the two aspects of OW [2], work community (18 items) [3], worker's resources and work (11 items) (1–5 Likert scale, 1 = totally disagree to 5 = totally agree). In addition, demographic information related to work and education was asked.

The semi‐structured interviews contained two parts: (1) experiences of the intervention relevance, including reflections on the main OW outcomes based on the Content Model ProSchoolSOWE [20, 21], and the role of the intervention in these outcomes, (2) Acceptability included attitude, usability, utility and engagement in the intervention based on the previous literature [32, 33, 34].

### 2.4 The Study Sample and Data Collection

The study used convenience sampling, selecting intervention and comparison work communities based on their willingness to participate and engage in a long intervention. The renewed instrument prevented conducting power analysis for an adequate sample size. The eligible study sample included all Finnish‐speaking health care educators working in health care programmes at two universities of applied sciences (intervention group *N* = 42, comparison group *N* = 70). The educators taught theoretical and/or practical components of nursing, public health nursing, midwifery or paramedic nursing. Surveys were sent at pre‐ and post‐test stages, with three reminders. Altogether 41 participants completed both tests (intervention *n* = 19, comparison *n* = 22; Figure 1). The groups were similar despite minor differences in demographic characteristics (Table 1) and represented the common distribution of Finnish health care educators in terms of the chosen backgrounds [5, 8].

**TABLE 1:** Demographics of the study participants.

	Intervention group *n* = 19	Comparison group *n* = 22
	Mean (SD)	Mean (SD)
Age in years	47.8 (8.4)	51.5 (7.2)
Work experience as an educator	9.0 (8.8)	12.7 (9.6)
Highest degree	F (%)	F (%)
Master's degree	18 (94.7)	18 (81.8)
Doctor's degree	1 (5.3)	4 (18.2)
Employment contract		
Permanent	15 (78.9)	21 (95.5)
Temporary	4 (21.1)	1 (4.5)
Remote work at least partially		
Yes	15 (78.9)	13 (59.1)
No	4 (21.1)	9 (40.9)

Abbreviations: F, frequency; SD, standard deviation (with permission from the authors).

Nine educators from the intervention work community were interviewed, either as OWE team members or general members of the work community. The number of interviews was based on data saturation. Interviews were conducted via Teams with sound recording, and one was face‐to‐face. They lasted between 31 and 62 min.

### 2.5 Data Analysis

Quantitative data were analysed using IBM SPSS Statistics 27. Based on previous factor analyses [5, 8], 29 items were grouped into six sum variables and assessed for internal consistency with Cronbach's alpha. Intervention outcomes were evaluated by within‐group and between‐group analyses using paired and independent samples T‐tests with effect size Cohen's *d*. Normal distribution was tested with the Shapiro–Wilk test, and bootstrapping of 100,000 samples was used for non‐normal data using R version 4.3.0. The data contained no significant outliers [39].

The qualitative interview data were analysed by deductive‐inductive content analysis (Graneheim et al. 2017; Lindgren et al. 2020). The Content Model ProSchoolSOWE [20, 21], acted as a deductive framework for analysing the intervention relevance on OW, while themes attitude, usability, utility and engagement [32, 33, 34], guided the acceptability analysis. Interview recording (7 h 21 min) was transcribed (97 pages) and units of analysis were coded according to the OW analysing framework and acceptability analysing framework. The coded unit of analysis (225 from OW, 170 from acceptability) was extracted in Microsoft Excel for Microsoft 365 program sheets. Within the frameworks, the analysis was then continued inductively, revealing subcategories (31 from OW, 34 from acceptability), categories (11 from OW, 13 from acceptability) and finally main categories (3 from OW, 4 from acceptability).

After analysing the intervention outcomes from both quantitative and qualitative methods, the main outcomes were created as quantitative and qualitative inferences. After which, the inferences were compared for similarities and differences, merged into mixed method inferences and finally into mixed method meta inferences, explaining and expanding the quantitative intervention outcomes [40, 41]. The main quantitative and qualitative outcomes and the confirming, expanding and discrepant mixed method meta‐inferences were reported through a joint display [42].

### 2.6 Ethical Considerations

This study followed the ethical principles for research according to the Declaration of Helsinki [43]. An ethical statement was issued by the UEF Committee on Research Ethics (7/2021 15.4.2021). Both participating educational institutions issued research approval for conducting the research. The participation was based on voluntariness, and all participants gave their informed electronic consent to participate in the study.

## 3 Results

### 3.1 Outcomes of the CBP‐OWE Intervention

Within‐ and between‐group comparison.

Overall self‐assessed level of personal and work community OW was evaluated as moderate to good at T0 and T1 with no significant changes between the timepoints. Within‐group comparison showed a significant increase in satisfaction in OW activities supported by the employer in the work community (Mean difference MD 1.16, ±0.78, *d* > 0.80, *p* < 0.001) and Workplace support (MD 0.58, ±0.55, *d* > 0.80, *p* < 0.001). These positive changes remained significant in between‐group comparisons (OW activities MD 1.13, *p* < 0.001; Workplace support MD 0.81, *p* < 0.001). OW actions related to Working arrangements (MD 0.13) and Management and information (MD 0.28) did not have significant change between the timepoints, and Collegiality and work atmosphere had decreased statistically significantly at T1 compared T0 (MD 0.39, ±0.54, *d =* 0.716, *p* < 0.01). The direction was positive in Resources and mental workload (MD 0.22, ±0.61), but the change between the timepoints was not statistically significant (Table 2).

**TABLE 2:** Within and between group comparison of OW in T0 and T1 timepoints.

Variable	Within IG (T0‐T1) (*n* = 19)			Within CG (T0‐T1) (*n* = 22)			Between IG‐CG	
	Mean SD T1/T0	Mean D SD (CI)	Effect^b^	Mean SD	Mean D SD (CI)	Effect^b^	Mean D^a^ (CI)	Effect^b^
OVERALL OW								
Personal OW^d^	T0 3.81 ± 0.77 T1 3.78 ± 0.87	0.02 ± 0.68 (−0.30, 0.35)	0.035	T0 4.00 ± 0.70 T1 3.85 ± 0.78	0.16 ± 0.69 (−0.15, 0.46)	0.232	‐0.14 (−0.57, 0.30)	0.198
Work community O^d^	T0 ^#^3.22 ± 0.67 T1^#^3.13 ± 0.70	0.09 ± 0.43 (−0.12, 0.30)	0.220	T0 3.47 ± 0.66 T1 3.35 ± 0.76	0.13 ± 0.74 (−0.20, 0.45)	0.168	‐0.03 (−0.43, 0.37)	0.051
OW ACTIVITIES								
OW activities employer^d^	T0 ^#^2.55 ± 0.80 T1 ^#^3.70 ± 0.63	**−1.16** *** ± 0.78 (−1.55, −0.76)	1.473	T0 3.13 ± 1.13 T1 3.15 ± 1.36	−0.03 ± 0.99 (−0.46, 0.41)	0.025	**‐1.13** *** (−1.71, −0.55)	1.249
OW activities leisure time^d^	T0 3.50 ± 1.01 T1 3.89 ± 0.90	−0.39‐ ± 1.00 ^c^(−0.75, 0.23)	0.467	T0 3.85 ± 0.89 T1 3.79 ± 1.08	0.05 ± 1.19 ^c^(−0.41, 0.65)	0.027	‐0.44 ^c^(−1.12, 0.27)	0.375
OW Aspects^e^								
Work community								
Management and information (6 items, α 0.805)	T0 ^#^3.14 ± 0.80 T1 ^#^2.86 ± 0.98	0.28 ± 0.78 (−0.11, 0.67)	0.355	^#^T0 3.83 ± 0.66 ^#^T1 3.83 ± 0.94	0.00 ± 0.73 (−0.33, 0.33)	0.000	0.28 (−0.21, 0.77)	0.368
Working arrangements (5 items, α 0.577)	T0 3.38 ± 0.67 T1 3.25 ± 0.82	0.13 ± 0.55 (−0.14, 0.39)	0.229	T0 3.68 ± 0.55 T1 3.67 ± 0.84	0.01 ± 0.57 ^c^(−0.21, 0.27)	0.002	0.12 ^c^(−0.24, 0.44)	0.206
Collegiality and work atmosphere (7 items, α 0.809)	T0 ^#^3.66 ± 0.79 T1 ^#^3.27 ± 0.82	**0.39** **± 0.54 (0.12, 0.66)	0.716	^#^T0 3.84 ± 0.46 ^#^T1 3.86 ± 0.75	−0.02 ± 0.72 (−0.37, 0.33)	0.031	0.41 (−0.02, 0.84)	0.640
Worker's resources and work								
Resources and mental workload (4 items, α 0.760)	T0 2.63 ± 0.89 T1 2.86 ± 1.04	−0.22 ± 0.61 (−0.52, 0.07)	0.369	^#^T0 3.22 ± 0.84 ^#^T1 3.09 ± 0.94	0.13 ± 0.62 (−0.15, 0.40)	0.201	‐0.35 (−0.74, 0.04)	0.568
Workplace support (4 items, α 0.542)	T0 2.82 ± 0.66 T1 3.39 ± 0.67	**−0.58** *** ±0.55 (−0.85, −0.31)	1.047	^#^T0 3.36 ± 0.71 ^#^T1 3.13 ± 0.91	0.23 ± 0.52 (−0.01, 0.46)	0.432	**‐0.81** *** (−1.15, −0.46)	1.497
Resources and physical workload (3 items, α 0.556)	T0 3.84 ± 0.86 T1 3.88 ± 0.79	−0.03 ± 0.69 ^c^(−0.32, 0.32)	0.080	T0 3.93 ± 0.72 T1 4.06 ± 0.66	−0.12 ± 0.59 ^c^(−0.42, 0.09)	0.187	0.09 ^c^(−0.30, 0.51)	0.129

Abbreviations: 95% CI, confidence interval of the difference, paired samples t‐test; CG, comparison group; IG, intervention group. (with permission from the authors).

^a^ Independent samples t‐test.

^b^ Cohen's *d* reported as the absolute value of the effect size.

^c^ Bootstrapping with 100,000 samples.

^d^ Scale 0–5.

^e^ Scale 1–5.

^#^ Missing information.

** Significant at *p* < 0.01 level.

*** Significant at *p* < 0.001 level.

### 3.2 Experiences of the Relevance of the Intervention on OW

The interviews revealed three main categories of experiences regarding the relevance of the on OW: (i) Overall OW and OW promoting activities, (ii) Intervention fostering work organisation and planning, (iii) Intervention promoting educators' resources (see Table S1). Educators' experiences of the intervention's relevance on the overall OW varied, and they considered the intervention as one form of OW promotion activity. Intervention fostering work organisation and planning included class schedule process, meeting arrangements, the balance between project work and teaching, and mentoring and orientation. Educators experienced that the intervention improved the class schedule process (objective 1.2; Figure 1) in terms of clear instructions, easy access to support, planning schedules, and clear and consistent processes, and meeting arrangements in terms of meeting invitations and accessible meeting agendas. The intervention increased discussion about the balance and systematic distribution of project work, but educators had conflicting experiences of the intervention's success in promoting balance between project work and teaching (objective 1.1; Figure 1) and in terms of utilising the received extra resources and balance level. Mentoring and orientation (objective 2; Figure 1) were seen as starting points for improvement; however, mentoring received less focus in the intervention actions.

Intervention promoting educators' resources included opportunities for work supervision, support for coping at work and OW promotion, and workload experiences. Most educators experienced that the intervention increased work supervision opportunities (objective 1.4; Figure 1), and they experienced increased support for coping at work, particularly through discussions about breaks and coping strategies. Educators had high workload experiences, and this intervention was not able to find solutions to backlogs and stressful periods of work.

### 3.3 Interview Experiences Explaining and Expanding the Pre‐ and Post‐Test Outcomes

The interview results confirmed and expanded the outcomes of the intervention but were partly discrepant (Table 3). The mixed‐method results indicate no change in overall OW experiences, but a positive change in OW‐promoting activities and workplace support for OW development during working hours. These positive changes included increased opportunities for work supervision, increased discussion and awareness about breaks and coping at work. Intervention and other development actions had a synergistic effect, bringing OW issues into joint discussion and a continuous agenda.

**TABLE 3:** Join display of key pre‐ and post‐test outcomes and interview experiences explaining and expanding the pre‐ and post‐test outcomes.

Theme	Pre‐ and post‐test outcomes: quantitative inferences	Interview experiences: qualitative inferences	Mixed‐method meta‐inferences
Overall OW	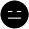 No significant changes	The varying perceived importance of intervention for overall OW	Explained: The interview results explained the insignificant results by diverse experiences of overall OW and the importance of the intervention on these experiences
OW promotion activities	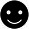 Significant increase Employer supported OW activities within group MD 1.16, *p* < 0.001; between group MD 1.13, *p* < 0.001	Intervention as a way to develop OW by making issues visible, under discussion and an active and continuous process. Intervention and other development activities support each other, but it is difficult to distinguish the intervention outcomes. Some considered the increase in OW development actions was due to the other development actions (like post‐COVID‐19 initiatives, sports and culture vouchers, joint activities)	Confirmed and expanded: The interview results explained that the increase in OW activities might stem from intervention, but also support other OW‐promoting activities. Intervention and other development actions had a synergistic effect and raised OW issues on the continuous agenda and under joint discussion
Work organisation and planning	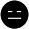 No significant change Working arrangements (such as working time arrangements, organisation of work and orientation; objectives 1.1 and 2; Figure 1)	Varying experiences regarding the balance between project work and teaching; some experiencing positive changes, others not a concrete change in the balance Increased discussion about balance More systematic, distributed working tasks Improved class schedule process Intervention initiated mentoring development; mentors were assigned to new employees, but no mentoring model, and the need for it was expressed	Confirmed and partly discrepant: Interview results explained the insignificant results of working arrangements by varying experiences of the actual balance between project work and teaching, although discussion about the topic's importance increased. Educators experienced considered intervention improved the class schedule process, being discrepant with quantitative results. Interview results explained the insignificant result of orientation by intervention, lacking the development of a mentoring model, although steps were taken towards it. Both results indicate the need to develop mentoring and orientation of new employees.
Information provision and decision‐making	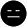 No significant change in Management and information (such as information and meetings) 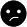 Significant decrease in Collegiality and work atmosphere MD 0.39, *p* < 0.01 such as open discussion; objective 1.3; Figure 1	Improved meeting arrangements: meeting invitations well in advance, easy access to meeting agendas Little experience, change in information and decision‐making and little implemented development actions	Confirmed and partly discrepant: The interview results explained the insignificant result in management and information and decreased Collegiality and work atmosphere, such as open discussion. Educators experienced intervention improved meeting arrangements, but little focus and implementing development actions for clear and open information and decision‐making, explaining the insignificant change and statistically significant decrease in ollegiality and work atmosphere
Workplace support for OW	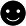 Significant increase Workplace support in withing group MD 0.58, *p* < 0.001 and between group MD 0.81, *p* < 0.001 (such as mental resources and coping at work, promoting OW during leisure time and working hours, work supervision; objective 1.4; Figure 1)	Intervention increased opportunities for work supervision Intervention, together with other OW actions in the organisation, increased discussion and awareness about breaks during working hours and coping at work. Other OW actions have been conducted to improve workspace ergonomics and break activity	Confirmed and expanded: Interview results explained the significant positive change in workplace support, by experiencing increased work supervision opportunities and increased discussion; however, it raises the value of other development activities performed during the intervention, which can partly explain the positive change.
Resources and mental workload	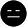 No significant increase in Resources and mental workload within group MD 0.22 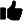 Direction was positive (such as balance of the mental workload, breaks and moments of rest)	The majority of interviewed educators considered the workload the same as before, and the intervention failed to solve the problem, although some considered steps towards a better balance between project work and teaching were taken	Confirmed: Interview results explained the insignificant positive change in workload by experienced workload, and the intervention failed to solve the problem, although the direction was positive

Abbreviations: MD, Mean difference; OW, occupational well‐being (with permission from the authors).

Community‐specific actions did not show statistically significant improvement on OW in the quantitative data, but the interviewed educators experienced improvements in work organisation processes such as class scheduling, meeting arrangements and reflection of OW issues. The objectives 1.3 Clear and open information and decision‐making and 2. Development of mentoring and orientation (Figure 1) received less attention, confirming the insignificant results. Although the intervention did not show statistically significant positive changes in experienced workload, there was a positive direction in balancing mental workload and breaks during working hours.

### 3.4 Acceptability of the CBP‐OWE Intervention

**FIGURE 2:**
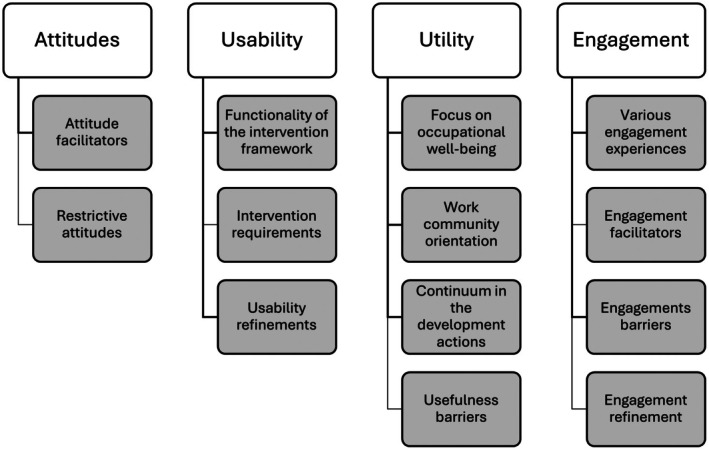
Acceptability of the intervention. (with permission from the authors).

Experiences of the acceptability of the intervention were expressed as attitudes, usability, utility, and engagement (Figure 2; Table S2). Attitude facilitators included interest in the intervention process, specific contents, and implementation methods such as OWE team meetings and joint discussions. Educators considered the intervention a positive experience and important. Restrictive attitudes were the pessimistic attitude within the work community towards the possibility of positive change in OW issues and experiences of disunity hindering community‐level development.

Usability was experienced as functionality of the framework, intervention requirements, and usability refinements. Educators considered the intervention framework clear and logical, with a focus on community‐specific needs. The OWE‐Edu course enabled participants to orient for OW development, the OWE team members were satisfied with the meeting content, working methods and received enough time resources for meetings and OW actions. Community‐specific development plans and actions were clearly structured and guided the process. The intervention required efforts to implement the planned development actions, keeping things visible in the work community and prioritising schedules to enable participation. Participation in the OWE‐Edu course required time from already busy schedules, where time resources for this were not. Usability refinements included a timely compact intervention with fewer objectives and allocated study time for the OWE‐Edu course.

Utility was experienced as a focus on OW, work community orientation and continuum in the development actions and usefulness barriers. The intervention process and OWE‐Edu course facilitated discussion, reflection, learning and development of personal and community OW. The work community orientation addressed community‐specific needs and goals, providing solutions for development needs raised. Continuum in the development actions referred to the intervention acting as a starting point for change, continuing an active process, with the possibilities to facilitate long‐term development processes. Expressed usefulness barriers included a lack of time and difficulty in detecting the effects of the long intervention.

Educators expressed engagement in terms of various engagement experiences, engagement facilitators, barriers and refinement. Some participated actively in the OWE teams, workshops, and meetings, while others experienced weak engagement, such as not completing the OWE‐Edu course and low participation in the meetings and development work. Engagement facilitators were described as interest and need for OW development, while barriers were experienced as feelings of being an outsider and a lack of information. Demanding work and time constraints also hindered engagement. Engagement refinements included better information provision, allocated time for the OWE‐Edu course and focusing on OW, and community‐level discussion and orientation events to support OW development.

## 4 Discussion

This study evaluated the outcomes and acceptability of the CBP‐OWE intervention among health care educators using quantitative, qualitative and mixed‐methods to explain and expand the pre‐ and post‐test outcomes and find suggestions for intervention refinement. The study showed success in OW promoting activities and workplace support for it. However, it lacked statistically significant results in overall OW experiences, which were one of the expected intervention outcomes.

Educators experienced with OW issues were kept on the agenda, discussed and taken seriously—they felt that management saw employees' OW as important and something to strive for. This emphasises the expected outcome of the intervention in terms of an OW supportive work community. An OW supportive work community is related to COH [26, 27], which should be emphasised in health‐promoting programmes [26]. COH can mediate intervention effectiveness [27], and thus support the expected long‐term intervention impact: the continuum of the OW development. Educators experienced OW development actions implemented in this intervention as an opportunity towards increased OW in the future. Positively experienced OW promoting activities relate to high OW experiences and may predict future progress [5].

This study used the Content Model ProSchoolSOWE for building and implementing the intervention. The model is based on resource and workload balance [22], a common approach in work‐related well‐being models such as the Job‐Demands‐Resources model [44]. Interventions addressing job demands at the organisation level, such as promoting work processes, are understudied [29]. This study aimed to improve several workload and work management issues by balancing project work, mentoring programmes and class scheduling, which had the most promising improvement. However, the CBP‐OWE intervention did not significantly change the experienced workload. Many studies have highlighted the heavy workload, backlog and poor work–life balance of health care educators [8, 9, 10, 45]. Reducing the workload and increasing work‐related resources of health care educational staff at the individual and organisational level requires sustainable and evidence‐based solutions to secure and maintain a competent health care workforce.

The other part of this study focused on intervention acceptability, relevant for the further development and applicability in educational practice [31, 32]. Based on the findings, the intervention forms a functional framework for community‐level OW development. Educators experienced the community‐specific focus in the development actions as especially increasing the intervention's usability, utility and engagement. Community‐level OW development actions should be based on community‐specific needs, with community members engaged in implementation and development actions [24]. The intervention components provided an evidence‐based framework [22, 25, 28, 30], where community‐specific OW actions and the OWE team, together with the work community interactions, were important factors for intervention engagement and the continuum of the OW development.

CBP‐OWE intervention involved participants at different community levels: employees, team leaders, and management, including OWE team members who were highly active in the community‐specific OW actions. Engagement levels varied within the work community and between OWE team members and regular members of the work community. Some participants, especially those not part of the OWE team, had experiences of being outsiders from the intervention and related information provision. Information, dialogue and communication are crucial throughout the process to enable involvement and engagement of the participants [24, 25]. This is not only crucial between the researchers and participants, but also between different levels of the work community. Emphasising the dialogue between management and employees acts as a buffer for organisational improvement and intervention success [24, 25].

Communication issues can also affect employee attitudes. Although attitudes towards the intervention were mostly positive, some experienced disunity and pessimistic attitudes towards change, which hindered the intervention's acceptability; decreased collegiality and work atmosphere were noticed based on quantitative results as well. Readiness for change is crucial to the success of the intervention, referring to the chain of effects starting from an attitude change, which is a functional enabler for the main expected outcome of improved well‐being [25]. Hence, it would have been important to study the participants' attitudes towards promoting OW, for example, in relation to the OWE‐Edu course participation. In addition, lack of time and demanding work seemed to be related to the engagement, usability and utility of the intervention, especially in the OWE‐Edu course participation; therefore, allocated time was suggested.

Evidence‐based methods are needed to promote OW and to respond to the shortage of health care educators. The CBP‐OWE intervention could be applied in health care educators' work communities to make organisational‐level improvements and to capture and seek solutions for actual causes of decreased OW. The authors suggest the following answers to the question of what works and under what circumstances: the components of the CBP‐OWE intervention work when participants are given enough time; adequate communication, dialogue and information about the progress are constant within the work community as well as between researchers and the work community; and the job demands allow educators' sufficient engagement in the intervention components and the implementation as a participatory intervention.

### 4.1 Strengths and Limitations

The strengths of the study were a quasi‐experimental pre‐ and post‐test intervention study design, use of mixed methods, and a validated instrument to measure intervention outcomes [38]. The trustworthiness of the qualitative analysis and mixed‐method data integration was increased by discussing the analysis process within the research group [42, 46]. However, the study contains several limitations that can limit the generalisation of the results. The sample size in both the intervention and comparison groups was small, which can affect the results. The relatively long period of the intervention posed limitations to revealing the intervention outcomes and keeping the participants engaged. Moreover, the change of the academic year during the intervention caused dropouts. Power analysis for the adequate sample size was impossible because the renewed instrument had not been used previously in intervention research; nevertheless, this study gives information for sample size calculations for future research. In addition, the internal consistency for some sum variables was below 0.6, indicating low internal consistency [39].

Changes in experienced OW take a long time [22, 25], and not very substantial changes can be expected in 1 year. The intervention was conducted during COVID‐19, which might also have affected the resources of the participating work community as well as social interactions. The participatory nature of the intervention limits following a strict plan and schedule in the intervention implementation [25], and separating intervention outcomes from other development activities that took place in the work community is difficult.

## 5 Conclusion

The CBP‐OWE intervention increased the experience of the work community and workplace support for OW actions but showed no change in the overall self‐assessed level of OW experiences. The intervention improved work processes and reflection on OW issues, and this can lead to longer term development work and improved OW experiences. The study acknowledges the complexity of the CBP‐OWE intervention process and the challenges of evaluating the intervention outcomes. This study suggests refining the intervention in terms of increasing the information, joint community‐level orientation, communication, and time resources for participants in the OWE‐Edu course and OW actions. The authors strongly recommend continuing to pursue community‐level OW intervention development; this approach allows organisation‐level solutions to be found for the actual causes of decreased OW, the development of structures, and enables the building of an OW supportive work community.

## Author Contributions

Anneli Vauhkonen, Kirsi Honkalampi, Jenni Rinne, Leena Salminen, Terhi Saaranen: conceptualisation, writing review, editing and methodology; Anneli Vauhkonen, Jenni Rinne, Terhi Saaranen: formal analysis software, validation, and visualisation; Anneli Vauhkonen, Kirsi Honkalampi, Terhi Saaranen: investigation and data curation; Anneli Vauhkonen, Jenni Rinne, Leena Salminen, Terhi Saaranen: funding acquisition; Terhi Saaranen, Kirsi Honkalampi: supervision; Terhi Saaranen: project administration. The statistical analysis has been checked by statistical expert Miko Pasanen.

## Funding

This work was supported by OAJ’s Occupational Wellbeing Fund.

## Ethics Statement

The ethical review of the study was conducted by the UEF Committee on Research Ethics (7/2021–15.4.2021), Finland.

## Consent

An electronic informed consent was obtained from all participants involved in the study.

## Conflicts of Interest

The authors declare no conflicts of interest.

## Supporting information


**Appendix S1:** Supporting Information.

**Appendix S2:** Supporting Information.

## Data Availability

The data are not publicly available due to privacy or ethical restrictions.
