# Supplementary material for: Outcomes and Acceptability of the Community‐Based Occupational Well‐Being Intervention Among Health Care Educators—Mixed Method Pilot Study
Source: Scand J Caring Sci. 2025 Dec 10;39(4):e70161. doi: 10.1111/scs.70161 (PMC12696404; doi:10.1111/scs.70161)
Supplement: Supplementary file 1 — Appendix S1: Supporting Information. [file SCS-39-0-s002.docx]

Supplementary material A: Supplementary Figure 1.


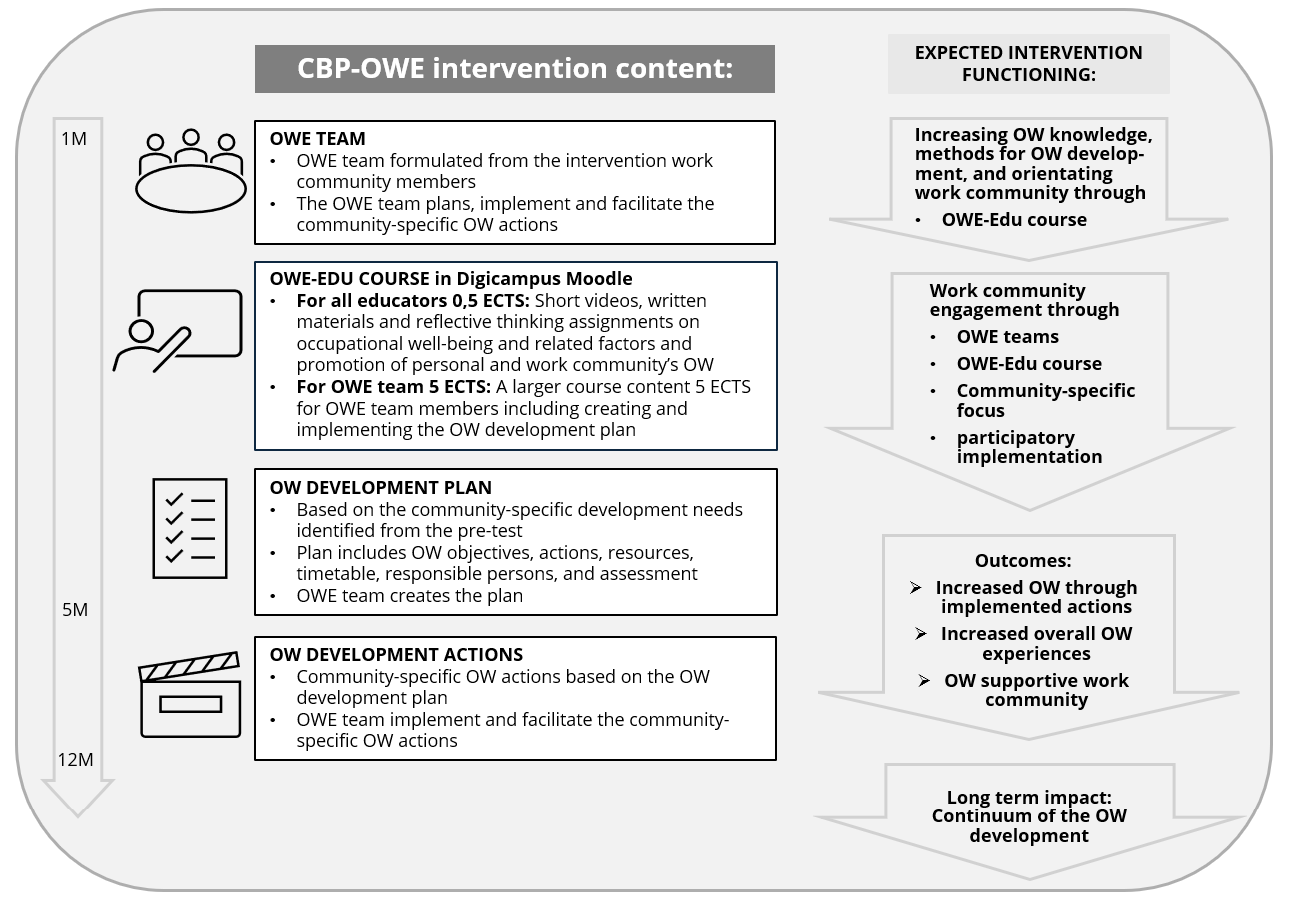


Supplementary Figure 1. Content and expected functionality of the CBP-OWE intervention. OWE team Occupational well-being team, OWE-Edu course Occupational WEll-being for Educators course, OW occupational well-being, M month. (with permission from the authors)
