# Supplementary material for: Outcomes and Acceptability of the Community‐Based Occupational Well‐Being Intervention Among Health Care Educators—Mixed Method Pilot Study
Source: Scand J Caring Sci. 2025 Dec 10;39(4):e70161. doi: 10.1111/scs.70161 (PMC12696404; doi:10.1111/scs.70161)
Supplement: Supplementary file 2 — Appendix S2: Supporting Information. [file SCS-39-0-s001.docx]

Supplementary material B: Supplementary Tables 1-2.

Supplementary Table 1. Categories and examples of expressions from the experienced relevance of the intervention on occupational well-being.

| **Main category** | **Category** | **Sub-category** | **Examples of the expressions (id of the participant)** |
| --- | --- | --- | --- |
| **Overall OW and OW promoting activities** | Varying perceived relevance on OW | Relevance on OW | “I was given the space and the opportunity to focus, so it was really important for me to be able to cope. And in terms of my productivity and occupational well-being... It was easier to breathe, let's put it this way. (i7) ” |
|  |  | No change on OW | “Beautiful and good ideas, but then again at that concrete level we haven't been able to get to the right challenges. (i1)” |
|  | Intervention as OW promoting activity | OW issues made visible | “Things have been made visible through the project and then when they are made visible it is easier to do something about them as well. That is, I could think that they could be related. (i6)” |
|  |  | Increasing discussion on OW promotion | “The project certainly had the effect that this occupational well-being issue and the activities supporting it were actively maintained in our discussion. I think it was handled much more actively and more often than before and I see that it is directly related to this project because there were these interventions. (i7)” |
|  |  | OW promotion as continuum process | “I would somehow imagine that this would also be a benefit that these things don't somehow just end. (i8)” |
|  | Merging intervention with other development activities | Supporting other development actions on OW | “Activities that increase the occupational well-being of the HR unit are going on, so they have, as it were, supported each other appropriately. i2) |
|  |  | Challenging to separate from other OW actions | “What is the result of this project, it is very difficult to separate it from that which is caused by or related to what. (i5)” |
|  |  | Relevance of other development activities | “I don't see either in these project goals and actions the connection to increasement of development actions. Perhaps more than that, because of the corona, some money may have been left unspent…(i4)” |
| **Intervention fostering work organisation and planning** | Class schedule process | Improving processes | “I can say from my point of view that it has improved and I am, at least, experiencing it as good. (i9)” |
|  |  | Clear and consistent process | “There has come like clarity and consistency in this. (i8)” |
|  |  | Schedule and calendar reminders | “And then like there are those reminder messages so at that point at the latest you will wake up like that this needs to be done now and class schedule. It works! (i3)” |
|  |  | Clear instructions and easy access support | “…lovely instructions, video instructions…really good instructions on what to do at what stage and so on. Class schedule channel in teams where you can ask questions. A lot of really good improvement have taken place. (i7)” |
|  |  | Join development of the process | “Since we had that core team of developers here, they have taken that message to their teams there and surely that's the general thinking in that class schedule process like evolved in the direction that this is like a thing to be developed together. (i1)” “Of course, there has been some kind of development work done beyond this development project (i4)” |
|  | Meeting arrangements | Meeting invitations in advance | “There has been an improvement in that now those invitations come, in a sense, for a longer period of time. (i9)” |
|  |  | Accessibility of the meeting agendas | “I think those meeting agendas have come more clearly with the meeting invitations. That one has better been able to familiarize them in advance than before. (i4)” |
|  | Balance between project work and teaching | Conflicting experiences of the balance | “It's like it's taken clear steps for the better. In my opinion, at least in my case. (i7)”; “Of course, there were resources, but nothing could be taken away from it... (i6)”; “…it's not still there that it actually feels like in the work... (i8)” |
|  |  | Increasing discussion about the balance | “I could imagine that the fact that things have been discussed so surely this project has had an impact…(i5)” |
|  |  | More systematically distributed working tasks | “Well, I'm sure it's going so much better that it's now that we have learned or noticed that like if everything does everything that it's not like the goal that everyone has to do everything but it's maybe better for people to be able to focus on something or some areas. (i7)” |
|  | Mentoring and orientation | Starting of the development work | “So, I have the impression that efforts are now done, and this is really taken as an issue and it's like an extremely good thing. (i8)”  “Of course, this (intervention) gave input to the fact that this was more accurately started (i2)” |
|  |  | Named mentor for new employees | “But I think it's like a definite improvement to the earlier, one being named and then that mentor gets working time so that you can actually sit down and talk. (i3)” |
|  |  | Lacking to achieve mentoring model | “No concrete change. In my opinion, we are in exactly the same situation as before the development project. (i4) “We haven't yet had some kind of agreement, or any content developed into what all this mentoring includes... (i2)” |
|  |  | Need for mentoring model | “It would be really a good if there would be some kind of structured model for the mentoring... (i9)” |
| **Intervention promoting educator’s resources** | Opportunity for work supervision | Increasing work supervision opportunities | “Yes it has improved considerably, it has been actively offered. And given so as opportunities are now announced as the latest in it's group work supervision. (i7)”  “Well, this is what it was like this brought it up, strengthening the earlier hopes, that this is where it came from, that there would be a wish to get work supervision. (i2)” |
|  |  | Work supervision opportunities contradicting the need | “The work supervision is available, so I feel that it is not. There is not such thing as corresponding the need, that there are some efforts, but it may not meet that need. (i3)” |
|  | Support for coping at work and OW promotion | Increasing discussion about breaks during working hours and coping at work | “I think that this kind of general discussion has increased from just taking a break from work and being able to cope with own work. (i1)”  “When the corona time came the organisation added information about taking care of own well-being and work-life balance. (i7)” |
|  |  | Awareness about coping at work | “So maybe that kind of awareness has increased and that's how it has had an impact on your everyday life. (i1)” |
|  |  | Additional supporting actions | “Sure, we have that e-pass for sport and it's supported like...supported to be used. Then some of these kinds of occupational well-being breaks have been arranged. (i3)”  “There's a little bit of some exercise bike and a pool table or a ping-pong table and some magnet hockey and a massage chair and all sorts of... and then the work ergonomics have been improved in those little offices. So these things support each other. (i1)” |
|  | Workload experiences | Heavy workload experiences | “I guess I can see that workload, it hasn't decreased. (i6)” |
|  |  | Backlog experiences | “I think the challenge is when the working tasks pile up in one spot. Whether it's looser at another point or not, it doesn't make it any easier at the point where those working tasks have piled up, that kind of like we haven't managed to solve it.” (i3) |
|  |  | Conflicting workload balance experiences | “Well, in terms of balance, that hasn't changed. (i3)”  “I guess it's like the worker's resources are affected by trying to balance that teaching and project work and those little lashes and blinks that people's workload consists of. Like looking at them as a whole… It's taken some clear steps for the better. At least in my case... (i7)” |
|  | Additional benefits of the intervention | Clean lab classrooms and hired instruments attendant | “The intervention was like an addition to it, like if you can say: in the screams that we need improvement (i2)” “It's been perhaps the best thing that's happened out there... My well-being has risen many times over for that person. (i9)” |

Note. OW occupational well-being. (with permission from the authors)

Supplementary Table 2. Categories and examples of expressions from the acceptability of the intervention.

| **Main category:** | **Category** | **Sub-category** | **Examples of the expressions (id of the participant)** |
| --- | --- | --- | --- |
| Attitude | Attitude facilitators | Interest towards intervention | “I felt like it was interesting to participate (i4).”; “interesting and to somehow slow down to think about those things… (i3)” |
|  |  | Positively perceived experience | “I have had a really positive experience with this (i1)” |
|  |  | Importance of the intervention | “I think this has been a great thing and something that needs to be done (i6)”; “I personally feel it is important that occupational well-being is developed (i1)”. |
|  | Restrictive attitudes | Pessimistic attitude towards change | “They felt that these things have been done and nothing ever changes (i1)” |
|  |  | Disunity and lack of team spirit | “We don't have the kind of team spirit or the kind of desire to do things together where we start developing as a group. (i7)” |
| Usability | Functionality of the intervention framework | Clear structure of the intervention | “This is how development actions should go, that they have, like, a clear path and there is the follow-up (i3).” |
|  |  | OWE-Edu course as orientation | “It was allowed to volunteer to participate in this online training, so in a way, when in your mind you already go to the world of occupational well-being. (i1)” |
|  |  | OWE team functionality | “Those meetings were good. They were very, very thorough, that they were done with effort, that enough time was spent on them. (i2)”; “It was always clear how to proceed and when to meet and so it worked. And the information was strong as well. (i5)” |
|  |  | Community specific development plan and actions | “This is where we clearly highlighted those things in our work community those...things that people get to influence themselves were allowed to tell us what's aching and what felt like this didn't work out and then actively sought out those solutions as well. (i1)” |
|  | Intervention requirements | Experienced efforts | “Of course, this requires that since things were being developed here, it was not just that I was answering the question, tick the box, but that I really had to think about how to handle this and then when I started doing something, it requires action. (i2)” It requires that things will be kept up. Otherwise, nothing will happen. (i1)” |
|  |  | Received time resources | “We had the resources to attend for these meetings… I'm sure I've got the resources for what's been going on here. (i1)”; “Well, compared to how much I participated in the meetings and development work and how much I got working resources for it, I think it responded. (i4)” |
|  | Usability refinements | Compact intervention | “Perhaps it would have been enough to have just one main objective because there were objectives underneath it as well. (i8); “What was now the outcomes of this intervention and what wasn't... as a timely compact package it could be easier to evaluate. (i5)” |
|  |  | Time resources | “About the online training that it should be like, where the time and place are created (i8)” |
| Utility | Focus on occupational well-being | Discussion and reflection on OW | “In a way, I get to reflect on things like this through a process like this, it was quite interesting. (i5)”. |
|  |  | Learning about OW | “That was really good the online course, you know, that there where things like that were familiar, but again things what you had to think like this is how it is, that we have to learn for ourselves. (i1)” “And in a way, it was something you could sort of commit to in your own life and think about, what this means to me, it was a kind of personal. (i1)”  “Then, of course, learn again about this process… (i2)” |
|  | Work community orientation | Community specific needs and goals | “Yes, in my opinion, the benefit itself is that we are involved in this kind of intervention research, which allows us to tell them about our own well-being, evaluate it and tell our wishes and to reflect on goals and think about how we would proceed to develop further. (i7)” |
|  |  | Solution-centred focus | “It was like the focus on the change that we want to achieve. (i3)” |
|  | Continuum in the development actions | Starting point for change | “We got good results here, I think, that we started to move forward… (i7)”; “The mentoring model is not finished, but nevertheless that there is a push forward from it. (i2)”; “At least try to make some changes, because even if that change doesn't immediately bring some change or satisfaction, then doing something like that really makes a big difference. (i8)” |
|  |  | Continuing and active process | “…active process, that it is such an existing continuous process that is not forgotten, not like there is some kind of an occupational well-being day for autumn and another day for spring and in between like nothing… (i7)” |
|  |  | Possibilities for long term development | “And it could stay alive so that it would be maintained. That's what I think and maybe I would see as my own area of responsibility so that…I could still maintain these themes in my own team and maybe in such a positive spirit. (i1)”;  “We would actually have to stop here as a team now. How do we want to develop and move forward? Well, that's actually the thing now, isn't it? Then it would go... then it might go on to life. (i6)” |
|  | Usefulness barriers | Lack of time | “If we had had enough time to put this here and to take over this, this would probably have produced stronger and better results. (i2)” |
|  |  | Difficult to distinguish the benefits | “My own main observation is that it is difficult to distinguish such a development activity as it were, from the rest of the activity. And it's kind of artificial, too. And of course, you might ask, well, maybe it's necessary because if that's how you think about the effect. Perhaps it would be good to be able to verify that it has been effective. (i5)” |
| Engagement | Various engagement experiences | Active engagement experiences | “I have been very active in all meetings and other things and have had discussions in my own team. (i5)”; “I have been involved in these meetings… we made those development plans together and we were also involved in development actions. (i7)” |
|  |  | Weak engagement experiences | “It frustrates regarding the development of occupational well-being, but it also frustrates the fact that we have been involved in such a development project so that I think we should have made a better commitment to this as a work community. (i4)” |
|  | Engagement facilitators | Interest in OW issues | “I'm interested in occupational well-being in general (i1)”; H4: Well, I guess that's kind of like the fact that when it comes to the theme of occupational well-being, it's important to me. |
|  |  | Need for OW development | “I guess the idea that it would be useful for my own work. (i5)”; “Well, the principle is that we now have something like this is great and we need it. (i6)” |
|  |  | Willingness to develop | “I am a bit of a developer that I want to be developing the work community and that workplace and my own occupational well-being and the well-being of others. And therefore, have seen this as important. (i6)” |
|  | Engagements barriers | Experiencing as outsider | “Of course, the challenge is probably that a lot of people are like on the outside. You know this kind of thing happens but you're in the outer circle. (i6)” |
|  |  | Weakness of information provision | “OWE team and its information… I didn't feel that it happened. (i7)” |
|  |  | Busy work | “What prevented you from going deeper into it, it's the hecticness of the job and all that… (i4)”; “At the same time I'm a little sad that when we're so busy we couldn't really jump right and invest into this. (i2)” |
|  | Engagement refinement | Increasing information | “Somehow modifying the communication with the idea that it would be kind of dynamic, that this has been done now, and the consequences are happening now, and that somehow, it would inspire people to participate. Like now some people read those newsletters that this is happening somewhere. (i2)” |
|  |  | More time | “It (OWE-Edu course) requires that there be such a long enough peaceful moment. (i3)”; “But it also requires that time…I mean that we would have this (OW) as a ceiling concept and have time, say, four hours to focus on it. (i6)” |
|  |  | Joint discussion | “…that we have time for that shared discussion and sharing. And maybe courage in a certain way as well. (i6)” |
|  |  | Orientation | “That there should be even more of these, even more of these kinds of four-hour sessions, where we would go through that OWE-Edu course material and somehow get the crowd to blow on the same coals first. (i1) |

Note. OW occupational well-being, OWE-Edu course Occupational WEll-being for Educators course, OWE team Occupational WEll-being development team. (with permission from the authors)
